# ATP Induces IL-1*β* Secretion in* Neisseria gonorrhoeae*-Infected Human Macrophages by a Mechanism Not Related to the NLRP3/ASC/Caspase-1 Axis

**DOI:** 10.1155/2016/1258504

**Published:** 2016-10-10

**Authors:** Killen García, Gisselle Escobar, Pablo Mendoza, Caroll Beltran, Claudio Perez, Sergio Arancibia, Rolando Vernal, Paula I. Rodas, Claudio Acuña-Castillo, Alejandro Escobar

**Affiliations:** ^1^Instituto de Investigación en Ciencias Odontológicas, Facultad de Odontología, Universidad de Chile, Santiago, Chile; ^2^Departamento de Gastroenterología, Hospital Clínico de la Universidad de Chile, Santiago, Chile; ^3^Banco de Sangre, Hospital Clínico de la Universidad de Chile, Santiago, Chile; ^4^Investigación y Desarrollo, Laboratorio Centrovet, Santiago, Chile; ^5^Departamento de Odontología Conservadora, Facultad de Odontología, Universidad de Chile, Santiago, Chile; ^6^Unidad de Odontología, Facultad de Ciencias de la Salud, Universidad Autónoma de Chile, Santiago, Chile; ^7^Center for Integrative Medicine and Innovative Science, Facultad de Medicina, Universidad Andrés Bello, Santiago, Chile; ^8^Laboratorio de Inmunología, Departamento de Biología, Facultad de Química y Biología, Universidad de Santiago de Chile, Santiago, Chile

## Abstract

*Neisseria gonorrhoeae* (Ngo) has developed multiple immune evasion mechanisms involving the innate and adaptive immune responses. Recent findings have reported that Ngo reduces the IL-1*β* secretion of infected human monocyte-derived macrophages (MDM). Here, we investigate the role of adenosine triphosphate (ATP) in production and release of IL-1*β* in Ngo-infected MDM. We found that the exposure of Ngo-infected MDM to ATP increases IL-1*β* levels about ten times compared with unexposed Ngo-infected MDM (*P* < 0.01). However, we did not observe any changes in inflammasome transcriptional activation of speck-like protein containing a caspase recruitment domain (CARD) (ASC, *P* > 0.05) and caspase-1 (CASP1, *P* > 0.05). In addition, ATP was not able to modify caspase-1 activity in Ngo-infected MDM but was able to increase pyroptosis (*P* > 0.01). Notably ATP treatment defined an increase of positive staining for IL-1*β* with a distinctive intracellular pattern of distribution. Collectively, these data demonstrate that ATP induces IL-1*β* secretion by a mechanism not related to the NLRP3/ASC/caspase-1 axis and likely is acting at the level of vesicle trafficking or pore formation.

## 1. Introduction

*Neisseria gonorrhoeae* (Ngo) or gonococcus, a Gram-negative diplococcus, is the etiological agent of the sexually transmitted bacterial infection (STI) gonorrhoea. Gonorrhea is the second most frequent STI worldwide, where the total number of new cases of gonorrhea in adults was estimated to be 106.1 million cases out of 498.9 million with STI [1]. Clinical data also indicates that previous gonococcal infections do not improve immune response in patients with reinfection, which suggest that immunological memory is not induced by gonococcus [2]. Since the ineffective immune response against gonococcus is multifactorial, it has been hypothesized that it could be the sum of different mechanisms. One of them may be related to genital tissue properties, such as immune privileged site in the female tract [3, 4], while the other could involve evasion mechanisms intrinsically developed by the bacteria, such as phase and antigenic variations [5], epitope mimicry [6], and phagosome subversion to overcome immune defence [7]. On the other hand, Ngo has several mechanisms for evading complement-mediated defences, such as LOS sialylation [8] and binding of PorB molecules to the complement cascade inhibitors factor H and complement factor 4b-binding protein (C4BP) [9].

The current data suggest that Ngo is able to suppress the protective innate cellular immune response at different levels. Even though phagocytosis by neutrophils (PMN) is generally a dead end for bacteria, it has been demonstrated that the number of intracellular Ngo cells and the number of bacteria per intracellular phagosome inside neutrophils increase over time [10–12]. Moreover, Ngo use multiple mechanisms to defend themselves against other ROS; nonoxidative antibacterial factors of PMN must act on the bacteria, which implies that these factors are not sufficient to clear infection [13]. On the other hand, macrophages (MΦ) and dendritic cells (DC) are critical cells in the innate immune response; it has been showed that Ngo potently inhibits the ability of antigen-primed bone-marrow-derived DC (BMDC) to trigger T-cell proliferation by inducing expression of both immunosuppressive cytokines and tolerance-inducing cell surface protein [14]. Recent findings of our laboratory have demonstrated that human monocyte-derived macrophages (MDM) were induced to a M2 profile when they were infected with Ngo. No significant differences were observed in IL-1*β* levels in comparison to gonococcus-treated macrophages and M0-macrophages suggesting that Ngo could trigger insufficient IL-1*β* levels to activate innate immune response, which leads to chronic inflammatory condition with no pathogen destruction [15].

IL-1*β* is described as a proinflammatory cytokine that is crucial for host-defense responses to infection and injury [16]. IL-1*β* needs two independent signals to be properly active and functionally released: a priming step where a first signal in response to pathogen associated molecular patterns (PAMP) induces pro-IL-1*β* expression [17] and a second signal which transforms pro-IL-1*β* in its mature and functional form (IL-1*β*) for proper release. This second signal is mediated by a protein complex named inflammasome, which prompts activation of the IL-1*β* converting enzyme caspase-1 [18], although it has been shown in other cells like PMN that production of IL-1*β* is not entirely dependent on caspase-1 and several serine proteases including CG, NE, and PR3 can also process pro-IL-1*β* [19].

In macrophages, multiple distinct bacterial products signals stimulate the NLRP3 inflammasome, resulting in the proteolytic activation of caspase-1 [20–22]. NLRP3 has a tripartite structure with a PAMP/danger associated molecular patterns (DAMP) sensing C-terminal leucine-rich repeat (LRR), a central nucleotide binding (or NACHT) domain, and an N-terminal pyrin domain (PYD) [23]. The PYD domain of NLRP3 recruits the adaptor molecule apoptosis-associated speck-like protein containing a caspase recruitment domain (ASC), which recruits pro-caspase-1 and produces caspase-1 activation [23].

Many pathogens have different mechanisms to disrupt inflammasome activation and therefore IL-1*β* production [24]. Thus, the searching for strategies to overcome this disruption is an important goal to be addressed. To date, three models of NLRP3 activation in response to microbial ligands have been proposed: K^+^ efflux, lysosomal rupture, and ROS production [25–27]. Additionally, endogenous DAMP such as extracellular ATP on macrophages produces a variety of cellular effects, including a maturation and release of IL-1*β* by inflammasome activation [28]. ATP can be secreted under a broad range of conditions including antigen presentation and macrophage interaction with bacteria or microbial products such as LPS [29].* In vitro* studies have demonstrated that ATP ligation of the P2X7 also stimulates killing of intracellular chlamydiae and mycobacteria in infected macrophages [30–32]. In addition, gingival epithelial cells infected with* P. gingivalis* secrete IL-1*β* only after stimulation with extracellular ATP [33]. Therefore, we tested the hypothesis that the addition of an exogenous source of ATP promotes the IL-1*β* processing and release during Ngo infection in human monocyte-derived macrophages (MDM). We demonstrated that ATP stimulation increases the secretion of IL-1*β* in Ngo-infected MDM. Interestingly, high levels of IL-1*β* did not correlate with the activation of inflammasome-mediated caspase-1, as previously observed in other microorganisms [33–35] suggesting that ATP-induced IL-1*β* secretion could be acting at the level of mechanisms related to vesicle trafficking or pore formation.

## 2. Materials and Methods

### 2.1. Blood Samples

Human buffy coats were provided by volunteers after informed consent. The study was approved by the Scientific Ethics Committee of Hospital Clínico Universidad de Chile (approval number 58). After sample collection, a trial number was assigned to each donor with their demographic data and the database was anonymised.

### 2.2. Bacteria and Culture Conditions

*Neisseria gonorrhoeae* P9-17 strain used in this study was kindly provided by Dr. Myron Christodoulides (University of Southampton, UK). Gonococcal vials were taken from frozen stocks, plated on Thayer Martin plates (LaboratorioLinsan, Chile), and cultured at 37°C in 5% CO_2_ for 18 to 20 hours. Single colonies with Pil^+^ Opa^+^ phenotype were reisolated for subsequent experiments.

### 2.3. Monocyte-Derived Macrophage (MDM) Generation

Human monocytes were obtained from normal blood donor buffy coats by two-step gradient centrifugation followed by an additional step using RosetteSep™ Human Monocyte Enrichment Cocktail (STEMCELL Technologies). Monocyte-derived macrophages (MDM) were obtained by culturing monocytes (84% CD14^+^) for 7 days in RPMI 1640 (GIBCO, Invitrogen Corporation) supplemented with 10% FBS (HyClone), 50 U/mL penicillin, 50 *μ*g/mL streptomycin (Gibco Invitrogen), and 50 ng/mL of M-CSF (MiltenyiBiotec) in 6-well plates at a density of 2 × 10^6^ cells per well.

### 2.4. ATP Solution Preparation

The necessary amount of ATP (product number A6419 Sigma-Aldrich, MO, USA) for experiment was weighed in a Eppendorf tube. Ten minutes before cells stimulation, ATP was diluted in nonsupplemented RPMI-1640 medium to generate a 10 mM ATP solution. Then ATP solution was filtered using syringe filter (pore size 0.2 *μ*m) and it was immediately added to the cells to reach a 5 mM concentration.

### 2.5. MDM Infection and Treatment with ATP

Bacterial suspensions were prepared in 1 mL serum-free RPMI1640 medium. MDM were infected with Ngo at a multiplicity of infection (MOI) of 100 for 4 hours at 37°C and 5% CO_2_. Then cultures were treated with gentamicin (100 *μ*g/mL) (Invitrogen Corp., Carlsbad, CA) to kill extracellular bacteria for 2 h at 37°C and 5% CO_2_. Medium was replaced by nonsupplemented (serum-free and antibiotic-free) RPMI and then 5 mM ATP (Sigma-Aldrich, MO, USA) was added and incubated for 30 minutes at 37°C 5% CO_2_. Finally, the supernatants were collected for IL-1*β*, while cells were lysed for RNA extraction.

### 2.6. Measurement of Secreted IL-1*β*

IL-1*β* levels were measured in supernatants immediately after 30-minute treatment with ATP by enzyme-linked immunosorbent assays using ELISA Ready-SET-Go! (eBioscience, USA, sensitivity 2 pg/mL), according to the manufacturer's instructions.

### 2.7. Quantitative Real-Time PCR (qRT-PCR)

Total RNA was extracted (purified) from MDM as described previously [36]. Reverse transcription (5 *μ*g) was performed using the Transcriptor First-Strand cDNA synthesis kit following the manufacturer's recommendations (Roche Applied Science, Mannheim, Germany). To quantify the mRNA expression of* IL-1β*,* NLRP3*,* ASC*, and* CASP-1*, 50 ng of cDNA was amplified by quantitative real-time PCR using the appropriate primers and the SybrGreen Master Mix (Fermentas) in a StepOne™ Real-Time PCR System (Applied Biosystems™, California, USA). The cycle program used was 95°C for 10 min, followed by 40 cycles of 95°C for 15 s, 60°C for 30 s, and 72°C for 30 s. Primer sequences were as follows: 5′-CTTCCTTTCCAGTTTGCTGC-3′ forward and 5′-TCTCGCAGTCCACTTCCTTT-3′ reverse for* NLRP3*, 5′-AGTTTCACACCAGCCTGGAA-3′ forward and 5′-TTTTCAAGCTGGCTTTTCGT-3′ reverse for* ASC*, 5′-GCCCAAGTTTGAAGGACAAA-3′ forward and 5′-GGTGTGGAAGAGCAGAAAGC-3′ reverse for* CASP-1*, 5′-CTGTCCTGCGTGTTGAAAGA-3′ forward and 5′-TTGGGTAATTTTTGGGATCTACA-3′ reverse for IL-1*β*, and 5′-CTAACACGGGAAACCTCAC-3′ forward and 5′-CGCTCCACCAACTAAGAACG-3′ reverse for 18S. The fold change in expression of the target gene relative to the 18S endogenous control was set at 2^−ΔΔCt^, where ΔΔCt = (CtTarget − Ct18S) stimulated − (CtTarget − Ct18S) unstimulated.

### 2.8. Detection of Enzymatically Active Caspase-1 and Apoptosis by Flow Cytometry

A FAM-FLICA caspase-1 assay kit (ImmunoChemistry Technologies) was used to detect caspase-1 enzymatic activity according to the manufacturer's instructions. FLICA was diluted and added to wells containing infected, ATP-treated or nontreated cells. The cells were cultured for 4 hours and MDM were then collected and subjected to flow-cytometric analysis (Facscalibur, Becton-Dickinson, San Diego, CA, USA) to quantify the percentage of FAM-FLICA-positive MDM. Apoptosis of MDM cells was measured using an FITC Annexin V Apoptosis Detection Kit with 7-AAD (BioLegend). Data analysis was performed using WinMDI 2.8 (freeware).

### 2.9. Immunofluorescence Microscopy Analysis

Macrophages were grown on 12 mm coverslips in 24-well plates and were treated using same conditions of cell culture previously described. After 30 minutes of ATP stimulation, cells supernatant was removed and coverslips were washed twice with sterile PBS 1x and then cells were fixed with 4% paraformaldehyde in 100 mM PIPES buffer pH 6.8, containing 40 mM KOH, 2 mM EGTA, and 2 mM MgCl_2_ for 30 minutes. Then, cells were washed three times with washing solution (50 mMTris buffer pH 7.6 containing 0.15 N NaCl and 0.1% sodium azide) and permeabilized with 0.1% Triton X-100 in washing solution for 10 min, washed twice, and then blocked with 2% bovine serum albumin for 60 min. Intracellular IL-1*β* was evaluated by staining cells with an anti-IL-1*β* Rb pAb at 1 : 100 dilution (ab2105, abcam, USA), followed by a goat anti-rabbit IgG (H+L) secondary antibody, Alexa Fluor® 488 conjugate (A11008, Life Technologies) at 1 : 100 dilution. Nuclear staining was carried out using DAPI (D9542 Sigma-Aldrich, St. Louis, MO). Coverslips were washed twice and mounted on microscope slides with DAKO fluorescent mounting medium (Dako North America Inc., CA, USA) and samples were visualized with a confocal microscopy (Carl Zeiss, model LSM700).

## 3. Results

### 3.1. *N. gonorrhoeae* Induces IL-1*β* Transcription but Inhibits IL-1*β* Secretion in MDM

**Figure 1:**
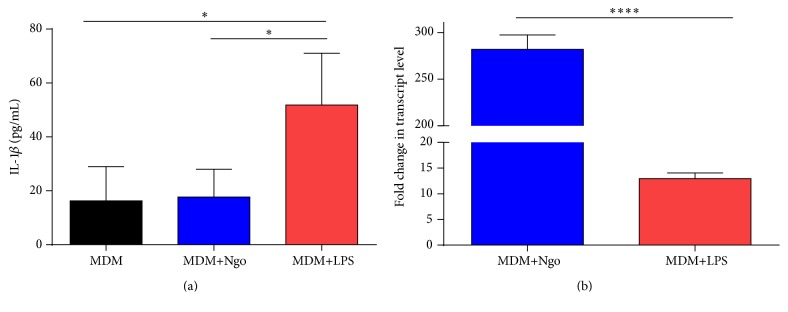
Ngo inhibits IL-1*β* levels in culture supernatants from infected MDM but is not able to inhibit IL-1*β* mRNA expression. MDM cells were infected with Ngo and then cytokine and RNA levels were evaluated after 30 minutes after infection. (a) Detection of IL-1*β* in culture supernatant by ELISA. (b) Transcriptional cytokine analysis by qRT-PCR. Results represent fold changes in transcript levels between no infected MDM and MDM infected with Ngo or treated with LPS. Data obtained are expressed as the mean ± SD and represent at least 6 independent experiments. ^*∗*^ *P* < 0.05; ^*∗∗∗∗*^ *P* < 0.0001.

Previous findings of our laboratory showed that IL-1*β* present in supernatants from Ngo- infected macrophages at different MOIs was not significantly different from nonstimulated MDM [15]. To further explore in those results, we aimed to detect the presence of IL-1*β* from MDM infected with Ngo at 100 MOI. As shown in Figure 1(a), macrophages infected with Ngo did not show more IL-1*β* secretion than noninfected macrophages. Indeed, IL-1*β* secretion by macrophages exposed to LPS was much more than IL-1*β* secreted by Ngo-infected macrophages. However, when total mRNA levels of pro-IL-1*β* were assessed by qPCR, we observed that pro-IL-1*β* RNA levels were highly induced in response to gonococcal infection (Figure 1(b)), indicating that Ngo is able to trigger the signaling pathway to start pro-IL-1*β* synthesis.

### 3.2. ATP Increases IL-1*β* Levels in Culture Supernatants from Ngo-Infected Macrophages

**Figure 2:**
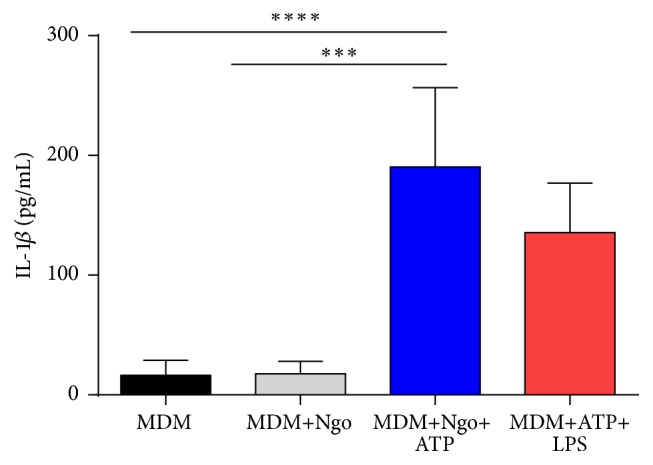
ATP treatment increases IL-1*β* levels by Ngo-infected MDM. MDM cells were infected with Ngo and then treated with ATP (5 mM) for 30 minutes. Culture supernatants were collected for measurement of IL-1*β* by ELISA. Data represent at least 4 independent experiments; bars indicate SD; ^*∗∗∗*^ *P* < 0.001; ^*∗∗∗∗*^ *P* < 0.0001.

Since the synthesis of mature IL-1*β* depends on the activation of NLRP3 inflammasome signaling pathway as a second signal [37], we aimed to stimulate the NLRP3 inflammasome in Ngo-infected MDM using a cognate agonist (ATP) [38], to establish whether gonococcus are modifying NLRP3 inflammasome activity. Our results showed that the low levels of secreted IL-1*β* from Ngo-infected MDM were increased in presence of ATP (Figure 2). The use of ATP was able to induce a similar cytokine production in Ngo-infected MDM as LPS stimulated MDM. It suggests that Ngo apparently is able to prevent inflammasome activation.

### 3.3. ATP Does Not Activate NLRP3 Inflammasome RNA Expression in Ngo-Infected MDM

Since one of the strategies adopted by bacterial pathogens is the inflammasome subversion [21] and the use of ATP contributes to the host immune response against intracellular bacteria, through its ability to stimulate P2X7-dependent IL-1*β* secretion [29], we decided to study whether inflammasome components* NLRP3*,* ASC*, and caspase-1 (*CASP-1*) might be modified by the ATP treatment in infected MDM. Data obtained from 4 independent experiments showed that ATP only modified the RNA expression levels of* NLRP3* in infected MDM, while the levels of* ASC* and* CASP-1* showed no significant differences with nonstimulated infected cells (Table 1).

### 3.4. ATP Does Not Modify Caspase-1 Activity in Ngo-Infected MDM but Is Able to Increase Pyroptosis

**Figure 3:**
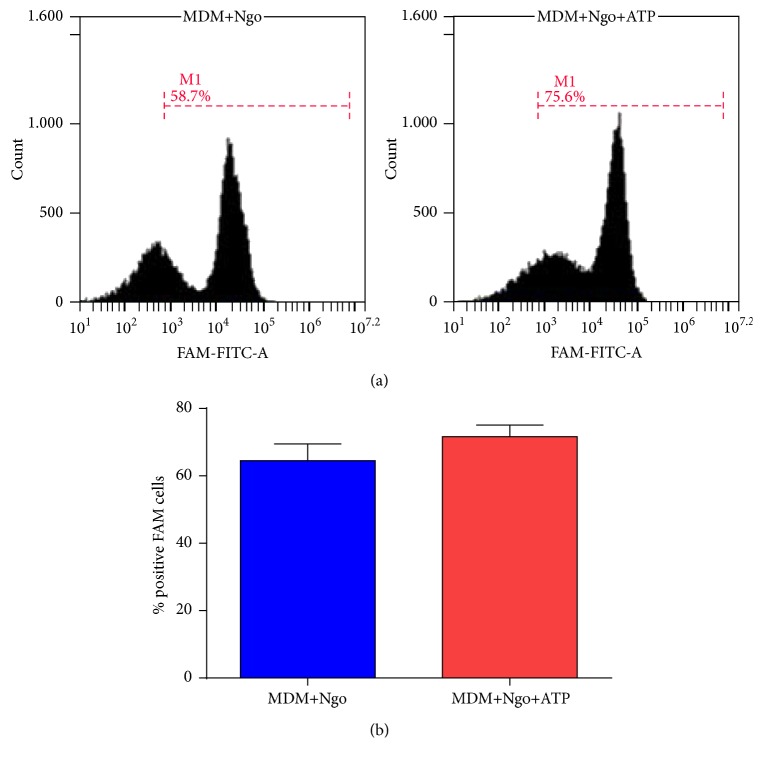
ATP treatment does not modify caspase-1 activity in Ngo-infected MDM. The fluorescent labeled probe of caspase-1 FAM-YVAD-FMK (FAM-FITC) was incubated with uninfected MDM, gonococcus-infected MDM (Ngo+MDM), or gonococcus-infected MDM and ATP (Ngo+MDM+ATP). After washing, the fluorescent intensity retained by the MDM was determined by flow cytometry. (a) Representative histograms from one assay are shown. (b) Percentage of positive FAM cells under different conditions. Data obtained are expressed as the mean ± SD and represent at least three independent experiments.

**Figure 4:**
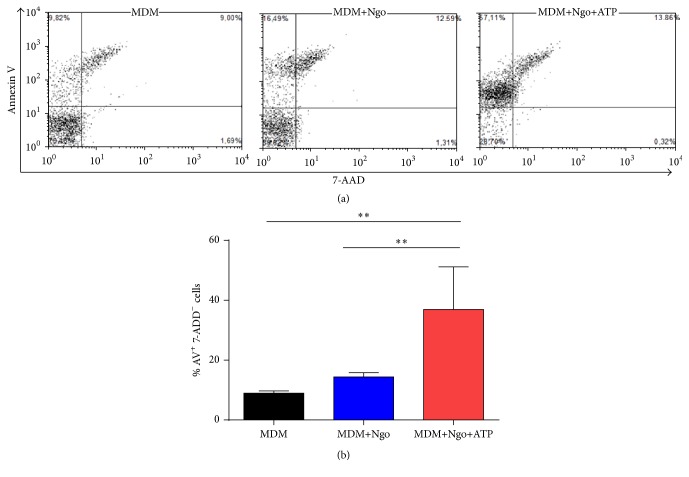
ATP treatment is able to increase apoptosis in Ngo-infected MDM. FITC Annexin V with 7-AAD staining was performed and the MDM population was determined by flow cytometry. (a) Representatives dot plot from one assay are shown. (b) Percentage of positive Annexin V and 7-AAD cells under different conditions. Data obtained are expressed as the mean ±  SD and represent at least three independent experiments. ^*∗∗*^ *P* < 0.01.

Caspase-1 activation is necessary to process pro-IL-1*β* onto mature IL-1*β*, but ATP was not able to produce transcriptional activation of* CASP-1*; thus we assessed if ATP is able to modify caspase-1 activity in Ngo-infected MDM, using a fluorescent inhibitor probe FAM-YVAD-FMK to label active caspase-1 enzyme. Data obtained from 3 independent experiments showed that ATP treatment of Ngo-infected MDM was not able to induce the activation of caspase-1 in infected cells (Figure 3). Activation of caspase-1 also triggers a form of cell death, known as pyroptosis [39]. Although trypan blue staining showed that most cells were viable after infection and ATP treatment, we performed a FITC Annexin V (AV) with 7-aminoactinomycin (7-AAD) Apoptosis Detection assay to monitor ATP-induced cell pyroptosis. Considering that the feature of pyroptosis is the positive staining with AV in contrast to negative 7-AAD staining [40], we compared the percentage of AV^+^ 7-AAD^−^ cells. ATP was able to induce an early stage of apoptosis in gonococcus-infected MDM (Figure 4).

### 3.5. ATP Affects the Intracellular Distribution of IL-1*β* in Ngo-Infected MDM Cells

**Figure 5:**
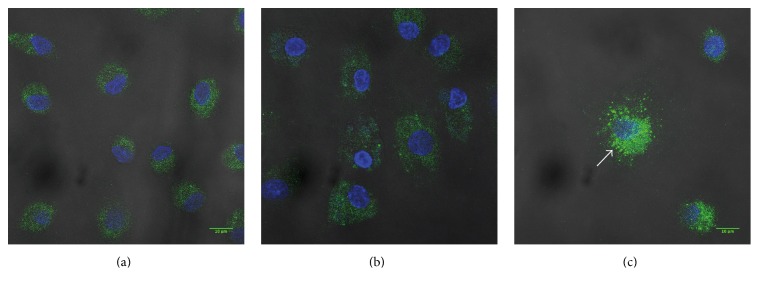
ATP modulates IL-1*β* distribution in Ngo-infected MDM cells. IL-1*β* expression was analyzed by confocal microscopy. (a) MDM cells, (b) Ngo-infected MDM. (c) Ngo-infected MDM treated with ATP. Images of IL-1*β* (green) and DAPI (blue). Arrow pointed IL-1*β* concentration. Scale bars 10 *μ*m.

Since NLRP3 inflammasome is not involved in the ATP-induced IL-1*β* secretion in gonococcus-infected MDM, we aimed to observe the ability of ATP to induce a nonclassical IL-1*β* secretion by microvesicles formation [41]. Ngo-infected MDM treated with ATP were observed for intracellular distribution of mature IL-1*β* by immunofluorescence using confocal microscopy. Notably, ATP treatment of Ngo-infected MDM defined an increase of positive staining for IL-1*β* with a distinctive pattern of distribution (Figure 5(c)) as a focus formation towards membrane.

## 4. Discussion

The current data suggest that Ngo produce lack of induction of protective immune response in macrophages and dendritic cell [14, 15, 42]. Recent findings of our laboratory have recognized that MDM are induced to a M2 profile when they are infected with Ngo and do not reach significant differences in IL-1*β* levels between gonococcus-treated macrophages and M0-macrophages [15]. In this work, we confirmed the lower levels of IL-1*β* in Ngo-infected human MDM (Figure 1(a)). Our data differ with those reported by Duncan et al. which showed that Ngo can promote NLRP3 activation and IL-1*β* secretion in human macrophages THP-1 cells [43]. This discrepancy might be explained by the remarkable difference between THP-1 cell line (monocytic nature) [44] and macrophages. The increase in the constitutive caspase-1 activation [45] and subsequent IL-1*β* secretion by monocytes following TLR stimulation is well recognized, whereas macrophages require a second signal [46]. Moreover, we showed that macrophages skewed from the M1 to M2 states, when infected with Ngo, have low levels of the Toll-like receptor-4 (TLR-4) [15], which has been shown to play a key role in the induction of inflammatory pathways [47].

The cytokine IL-1*β* is controlled by two checkpoints: transcription and maturation and release [48]. Our first results showed lower levels of mature IL-1*β* in Ngo-infected human MDM supernatants. However, when we looked at transcript level, we showed a sizable induction of IL-1*β* RNA upon gonococcal infection (Figure 1(b)). The differences in transcription versus translation of IL-1*β* induced by gonococcus in MDM cells could be due to disruption in inflammasome activity as shown in others pathogens [20]. Since ATP contributes to the host immune response against intracellular bacteria through its ability to stimulate IL-1*β* secretion [29], we showed that ATP treatment of gonococcus-infected MDM cells is able to restore IL-1*β* secretion (Figure 2) as shown in gingival epithelial cells infected with* P. gingivalis*, which only secrete IL-1*β* after stimulation with extracellular ATP [33]. After having established the effect of ATP on IL-1*β* production by gonococcus-treated MDM, we examined the mRNA expression of NLRP3 inflammasome-associated genes:* NLRP3*,* ASC*, and* CASP-1* to assess inflammasome transcriptional activity in presence of ATP. Only the* NLRP3* transcript was upregulated in Ngo-infected MDM cells treated with ATP (Table 1). To date, the exact action mechanism of ATP on transcription of inflammasome is unknown. However, it is not surprising that expression of* NLRP3* was affected by ATP, because the ATP release may act as a danger signal to mobilize intracellular calcium, further modulating NF-*κ*B activation acting through a P2Y receptor/phospholipase-C/calcium signaling pathway [49]. In this context, the existence of binding sequences to NF-*κ*B has been demonstrated in the NLRP3 promoter in murine macrophages [50].

Interestingly, we did not detect any changes in transcription levels of* ASC* and* CASP-1* in Ngo-infected macrophages treated with ATP (Table 1). The nonchange in levels of expression of* ASC* and* CASP-1* could be explained because unlike NLRP3, its transcriptional regulation is not associated with MyD88 and NF-*κ*B [51]. In line with this, we think that the absence of changes in transcription level of ASC could be affecting caspase-1 activity in response to ATP, because the function of adaptor protein ASC is enabling the activation of caspase-1 in inflammasome. Indeed, Asc^−/−^ macrophages secreted much less mature IL-1*β* than their wild-type counterparts in* S. typhimurium*-induced caspase-1 activation model [52].

Although we did not observe changes in transcription levels of CASP-1, we assessed the caspase-1 activity in Ngo-infected MDM cells treated with ATP. We did not observe any changes in caspase-1 activity between infected MDM cells with or without ATP. Interestingly, in our model of MDM cells, infection with Ngo does not induce pyroptosis compared with uninfected MDM cells, unlike what is reported by Duncan et al., who showed that Ngo infection promotes NLRP3-dependent THP-1 cell death via pyronecrosis [43]. However, when infected MDM cells were treated with ATP, we observed an induction of early apoptotic cells (AV^+^ 7AAD^−^). Even though pyroptosis is caspase-1-dependent, we do not discard other mechanisms that induce apoptosis. As showed by previous works, extracellular ATP induces apoptotic signaling in different cells types using different pathways that link purinergic receptors to induce apoptosis and necrosis [53–55].

Our results showed that ATP was not able to activate caspase-1 at RNA and protein level in Ngo-infected macrophages (Table 1 and Figure 3). Since pro-IL-1*β* and pro-IL-18 are the substrates* in vitro* and* in vivo* of caspase-1 [56], we hypothesize that IL-1*β* secretion induced by ATP in Ngo-infected macrophages could be at an alternative level from pro-IL-1*β* processing by NLRP3 inflammasome. In a recent report studying* Porphyromonas gingivalis-*infected mice macrophages, IL-1*β* secretion was inhibited by the pathogen fimbriae through inflammasome independent P2X7R mechanisms, suggesting that, in this pathological context, the IL-1*β* absence was due to unknown secretory signaling pathway involving P2X7R [57].

Using confocal microscopy, we attempted to localize the presence and distribution of mature IL-1*β*, where it was clearly visible that Ngo-infected MDM induced a pattern of the mature IL-1*β* arrangement with stained points dispersed in the cells. This dispersion of mature IL-1*β* as stained points was changed to concentrated and polarized points (Figure 5). Since the secretion mechanism to IL-1*β* does not correspond to the classic cytokine ER-Golgi pathway [58] and its secretion could be carried by intracellular vesicles, secretion lysosome exocytosis, or direct passing through the cellular membrane [41], we speculate that one of the possibilities in the downmodulation of IL-1*β* secretion by gonococcus could be due to a mechanism related to pore formation. Indeed, during pyroptosis, pores open in the cell membrane and this structural change could promote the cellular release of interleukin-1*β* [39]. Moreover, as shown by Pelegrin and Surprenant, pannexin-1 (Panx1) could be a functional link between P2X7R-activated large pore formation and cytokine release in macrophage. In this sense, the activation of P2X7-receptor triggers the recruitment of an accessory pore-forming mechanism [59, 60]; the Panx1 protein knockdown and panx1-mimetic inhibitory peptide are able to block ATP-mediated IL-1*β* release in mouse and human macrophage [61]. This issue was not assessed in this work and further studies are required to investigate this phenomenon.

## 5. Conclusion

In this work, we demonstrated that* N. gonorrhoeae* probably evades host defence by not activating the NLRP3 inflammasome in macrophages and showed that extracellular ATP is likely to act at the level of vesicle trafficking or pore formation. A better understanding of mechanisms that produce the IL-1*β* downmodulation by gonococcus will further contribute to the advance of our knowledge on* N. gonorrhoeae* pathogenesis and let us find a therapeutic strategy for treatment of gonococcus infection.

## Figures and Tables

**Table 1:** Transcriptional activation of inflammasome-associated genes in MDM.

Gene designation	Protein	Fold change^a^	
		MDM+Ngo	MDM+Ngo+ATP
*NLRP3*	NLR family, pyrin domain- containing protein 3	0.67	3.45^†^
*ASC*	Apoptosis-associated speck-like protein containing a caspase recruitment domain	0.54	0.63
*CASP1*	Caspase-1	2.1	2.6

^a^Values represent fold changes in transcript levels between MDM infected with *Neisseria gonorrhoeae* and MDM infected with *Neisseria gonorrhoeae* treated with ATP. ^†^ *P* < 0.05. Mann–Whitney *U* test.

## References

[1] WHO (2012). *Global Incidence and Prevalence of Selected Curable Sexually Transmitted Infections-2008*.

[2] Hedges S. R., Mayo M. S., Mestecky J., Hook E. W., Russell M. W. (1999). Limited local and systemic antibody responses to Neisseria gonorrhoeae during uncomplicated genital infections. *Infection and Immunity*.

[3] Imarai M., Candia E., Rodriguez-Tirado C. (2008). Regulatory T cells are locally induced during intravaginal infection of mice with Neisseria gonorrhoeae. *Infection and Immunity*.

[4] Niederkorn J. Y. (2006). See no evil, hear no evil, do no evil: the lessons of immune privilege. *Nature Immunology*.

[5] van der Woude M. W., Bäumler A. J. (2004). Phase and antigenic variation in bacteria. *Clinical Microbiology Reviews*.

[6] Harvey H. A., Swords W. E., Apicella M. A. (2001). The mimicry of human glycolipids and glycosphingolipids by the lipooligosaccharides of pathogenic *Neisseria* and *Haemophilus*. *Journal of Autoimmunity*.

[7] Billker O., Popp A., Brinkmann V. (2002). Distinct mechanisms of internalization of Neisseria gonorrhoeae by members of the CEACAM receptor family involving Rac1- and Cdc42-dependent and -independent pathways. *EMBO Journal*.

[8] Gill M. J., Mcquillen D. P., Van Putten J. P. M. (1996). Functional characterization of a sialyltransferase-deficient mutant of Neisseria gonorrhoeae. *Infection and Immunity*.

[9] Ram S., McQuillen D. P., Gulati S., Elkins C., Pangburn M. K., Rice P. A. (1998). Binding of complement factor H to loop 5 of porin protein 1A: a molecular mechanism of serum resistance of nonsialylated *Neisseria gonorrhoeae*. *The Journal of Experimental Medicine*.

[10] Casey S. G., Veale D. R., Smith H. (1979). Demonstration of intracellular growth of gonococci in human phagocytes using spectinomycin to kill extracellular organisms. *Journal of General Microbiology*.

[11] Veale D. R., Goldner M., Penn C. W., Ward J., Smith H. (1979). The intracellular survival and growth of gonococci in human phagocytes. *Journal of General Microbiology*.

[12] Casey S. G., Shafer W. M., Spitznagel J. K. (1986). Neisseria gonorrhoeae survive intraleukocytic oxygen-independent antimicrobial capacities of anaerobic and aerobic granulocytes in the presence of pyocin lethal for extracellular gonococci. *Infection and Immunity*.

[13] Criss A. K., Seifert H. S. (2012). A bacterial siren song: intimate interactions between Neisseria and neutrophils. *Nature Reviews Microbiology*.

[14] Zhu W., Ventevogel M. S., Knilans K. J. (2012). Neisseria gonorrhoeae suppresses dendritic cell-induced, antigen-dependent CD4 T cell proliferation. *PLoS ONE*.

[15] Ortiz M. C., Lefimil C., Rodas P. I. (2015). *Neisseria gonorrhoeae* modulates immunity by polarizing human macrophages to *α* M2 profile. *PLoS ONE*.

[16] Lopez-Castejon G., Brough D. (2011). Understanding the mechanism of IL-1*β* secretion. *Cytokine and Growth Factor Reviews*.

[17] Takeuchi O., Akira S. (2010). Pattern recognition receptors and inflammation. *Cell*.

[18] Schroder K., Tschopp J. (2010). The inflammasomes. *Cell*.

[19] Guma M., Ronacher L., Liu-Bryan R., Takai S., Karin M., Corr M. (2009). Caspase 1-independent activation of interleukin-1*β* in neutrophil-predominant inflammation. *Arthritis & Rheumatism*.

[20] Yang C.-S., Shin D.-M., Jo E.-K. (2012). The role of NLR-related protein 3 inflammasome in host defense and inflammatory diseases. *International Neurourology Journal*.

[21] Anand P. K., Malireddi R. K. S., Kanneganti T.-D. (2011). Role of the Nlrp3 inflammasome in microbial infection. *Frontiers in Microbiology*.

[22] Hise A. G., Tomalka J., Ganesan S. (2009). An essential role for the NLRP3 inflammasome in host defense against the human fungal pathogen *Candida albicans*. *Cell Host and Microbe*.

[23] Mariathasan S. (2007). ASC, Ipaf and Cryopyrin/Nalp3: bona fide intracellular adapters of the caspase-1 inflammasome. *Microbes and Infection*.

[24] Rathinam V. A. K., Vanaja S. K., Fitzgerald K. A. (2012). Regulation of inflammasome signaling. *Nature Immunology*.

[25] Davis B. K., Wen H., Ting J. P.-Y. (2011). The Inflammasome NLRs in immunity, inflammation, and associated diseases. *Annual Review of Immunology*.

[26] Pétrilli V., Papin S., Dostert C., Mayor A., Martinon F., Tschopp J. (2007). Activation of the NALP3 inflammasome is triggered by low intracellular potassium concentration. *Cell Death and Differentiation*.

[27] Zhou R., Yazdi A. S., Menu P., Tschopp J. (2011). A role for mitochondria in NLRP3 inflammasome activation. *Nature*.

[28] Yilmaz Ö., Yao L., Maeda K. (2008). ATP scavenging by the intracellular pathogen Porphyromonas gingivalis inhibits P2X7-mediated host-cell apoptosis. *Cellular Microbiology*.

[29] Coutinho-Silva R., Ojcius D. M. (2012). Role of extracellular nucleotides in the immune response against intracellular bacteria and protozoan parasites. *Microbes and Infection*.

[30] Coutinho-Silva R., Stahl L., Raymond M. N. (2003). Inhibition of chlamydial infectious activity due to P2X7R-dependent phospholipase D activation. *Immunity*.

[31] Darville T., Welter-Stahl L., Cruz C., Sater A. A., Andrews C. W., Ojcius D. M. (2007). Effect of the purinergic receptor P2X7 on Chlamydia infection in cervical epithelial cells and vaginally infected mice. *Journal of Immunology*.

[32] Fairbairn I. P., Stober C. B., Kumararatne D. S., Lammas D. A. (2001). ATP-mediated killing of intracellular mycobacteria by macrophages is a P2X7-dependent process inducing bacterial death by phagosome-lysosome fusion. *Journal of Immunology*.

[33] Yilmaz Ö., Sater A. A., Yao L., Koutouzis T., Pettengill M., Ojcius D. M. (2010). ATP-dependent activation of an inflammasome in primary gingival epithelial cells infected by *Porphyromonas gingivalis*. *Cellular Microbiology*.

[34] Ali S. R., Timmer A. M., Bilgrami S. (2011). Anthrax toxin induces macrophage death by p38 MAPK inhibition but leads to inflammasome activation via ATP leakage. *Immunity*.

[35] Kelk P., Abd H., Claesson R., Sandström G., Sjöstedt A., Johansson A. (2011). Cellular and molecular response of human macrophages exposed to *Aggregatibacter actinomycetemcomitans* leukotoxin. *Cell Death and Disease*.

[36] Vernal R., Velásquez E., Gamonal J., Garcia-Sanz J. A., Silva A., Sanz M. (2008). Expression of proinflammatory cytokines in osteoarthritis of the temporomandibular joint. *Archives of Oral Biology*.

[37] Haneklaus M., O'Neill L. A. J. (2015). NLRP3 at the interface of metabolism and inflammation. *Immunological Reviews*.

[38] Gombault A., Baron L., Couillin I. (2012). ATP release and purinergic signaling in NLRP3 inflammasome activation. *Frontiers in Immunology*.

[39] Miao E. A., Leaf I. A., Treuting P. M. (2010). Caspase-1-induced pyroptosis is an innate immune effector mechanism against intracellular bacteria. *Nature Immunology*.

[40] Miao E. A., Rajan J. V., Aderem A. (2011). Caspase-1-induced pyroptotic cell death. *Immunological Reviews*.

[41] Piccioli P., Rubartelli A. (2013). The secretion of IL-1*β* and options for release. *Seminars in Immunology*.

[42] Escobar A., Candia E., Reyes-Cerpa S. (2013). *Neisseria gonorrhoeae* induces a tolerogenic phenotype in macrophages to modulate host immunity. *Mediators of Inflammation*.

[43] Duncan J. A., Gao X., Huang M. T.-H. (2009). *Neisseria gonorrhoeae* activates the proteinase cathepsin B to mediate the signaling activities of the NLRP3 and ASC-containing inflammasome. *The Journal of Immunology*.

[44] Tsuchiya S., Yamabe M., Yamaguchi Y., Kobayashi Y., Konno T., Tada K. (1980). Establishment and characterization of a human acute monocytic leukemia cell line (THP-1). *International Journal of Cancer*.

[45] Netea M. G., Nold-Petry C. A., Nold M. F. (2009). Differential requirement for the activation of the inflammasome for processing and release of IL-1*β* in monocytes and macrophages. *Blood*.

[46] Burchett S. K., Weaver W. M., Westall J. A., Larsen A., Kronheim S., Wilson C. B. (1988). Regulation of tumor necrosis factor/cachectin and IL-1 secretion in human mononuclear phagocytes. *Journal of Immunology*.

[47] Liu M., John C. M., Jarvis G. A. (2010). Phosphoryl moieties of lipid a from *Neisseria meningitidis* and *N. gonorrhoeae* lipooligosaccharides play an important role in activation of both MyD88-and TRIF-dependent TLR4-MD-2 signaling pathways. *Journal of Immunology*.

[48] Dinarello C. A. (2009). Immunological and inflammatory functions of the interleukin-1 family. *Annual Review of Immunology*.

[49] De Oliveira S., López-Muñoz A., Candel S., Pelegrín P., Calado Â., Mulero V. (2014). ATP modulates acute inflammation in vivo through dual oxidase 1-derived H_2_O_2_ production and NF-*κ*B activation. *Journal of Immunology*.

[50] Qiao Y., Wang P., Qi J., Zhang L., Gao C. (2012). TLR-induced NF-*κ*B activation regulates NLRP3 expression in murine macrophages. *FEBS Letters*.

[51] Zhang A., Wang P., Ma X. (2015). Mechanisms that lead to the regulation of NLRP3 inflammasome expression and activation in human dental pulp fibroblasts. *Molecular Immunology*.

[52] Mariathasan S., Newton K., Monack D. M. (2004). Differential activation of the inflammasome by caspase-1 adaptors ASC and Ipaf. *Nature*.

[53] Costa-Junior H. M., Mendes A. N., Davis G. H. N. G. (2009). ATP-induced apoptosis involves a Ca^2+^-independent phospholipase A2 and 5-lipoxygenase in macrophages. *Prostaglandins and Other Lipid Mediators*.

[54] Yoon M.-J., Lee H.-J., Kim J.-H., Kim D.-K. (2006). Extracellular ATP induces apoptotic signaling in human monocyte leukemic cells, HL-60 and F-36P. *Archives of Pharmacal Research*.

[55] Zheng L. M., Zychlinsky A., Liu C.-C., Ojcius D. M., Young J. D.-E. (1991). Extracellular ATP as a trigger for apoptosis or programmed cell death. *The Journal of Cell Biology*.

[56] Sollberger G., Strittmatter G. E., Garstkiewicz M., Sand J., Beer H.-D. (2014). Caspase-1: the inflammasome and beyond. *Innate Immunity*.

[57] Ramos-Junior E. S., Morandini A. C., Almeida-Da-Silva C. L. C. (2015). A Dual role for P2X7 Receptor during *Porphyromonas gingivalisInfection*. *Journal of Dental Research*.

[58] Schatz G., Dobberstein B. (1996). Common principles of protein translocation across membranes. *Science*.

[59] Faria R. X., DeFarias F. P., Alves L. A. (2005). Are second messengers crucial for opening the pore associated with P2X 7 receptor?. *American Journal of Physiology—Cell Physiology*.

[60] Schilling W. P., Wasylyna T., Dubyak G. R., Humphreys B. D., Sinkins W. G. (1999). Maitotoxin and P2Z/P2X_7_ purinergic receptor stimulation activate a common cytolytic pore. *American Journal of Physiology—Cell Physiology*.

[61] Pelegrin P., Surprenant A. (2006). Pannexin-1 mediates large pore formation and interleukin-1*β* release by the ATP-gated P2X7 receptor. *The EMBO Journal*.

